# The Effects of Synbiotics Administration on Stress-Related Parameters in Thai Subjects—A Preliminary Study

**DOI:** 10.3390/foods11050759

**Published:** 2022-03-06

**Authors:** Ekasit Lalitsuradej, Sasithorn Sirilun, Phakkharawat Sittiprapaporn, Bhagavathi Sundaram Sivamaruthi, Komsak Pintha, Payungsak Tantipaiboonwong, Suchanat Khongtan, Pranom Fukngoen, Sartjin Peerajan, Chaiyavat Chaiyasut

**Affiliations:** 1 Innovation Center for Holistic Health, Nutraceuticals, and Cosmeceuticals, Chiang Mai University, Chiang Mai 50200, Thailand; ekasit_l@cmu.ac.th (E.L.); sivamaruthi.b@cmu.ac.th (B.S.S.); suchanat_k@cmu.ac.th (S.K.); pranom_fukngoen@cmu.ac.th (P.F.); 2 Department of Pharmaceutical Sciences, Faculty of Pharmacy, Chiang Mai University, Chiang Mai 50200, Thailand; 3 Neuropsychological Research Laboratory, Department of Anti-Aging and Regenerative Science, School of Anti-Aging and Regenerative Medicine, Mae Fah Luang University, Bangkok 10110, Thailand; 4 Office of Research Administration, Chiang Mai 50200, Thailand; 5 Department of Biochemistry, Faculty of Medical Science, University of Phayao, Phayao 56000, Thailand; komsakjo@gmail.com (K.P.); payungsak.t@gmail.com (P.T.); 6 Health Innovation Institute, Chiang Mai 50200, Thailand; s.peerajan@gmail.com

**Keywords:** synbiotics, stress, *Lactobacillus*, *Bifidobacterium*, galacto-oligosaccharides, oligofructose

## Abstract

Urbanization influences our lifestyle, especially in fast-paced environments where we are more prone to stress. Stress management is considered advantageous in terms of longevity. The use of probiotics for psychological treatment has a small amount of diverse proven evidence to support this. However, studies on stress management in stressed subjects using synbiotics are still limited. The present study aimed to investigate the effects of synbiotics on stress in the Thai population. A total of 32 volunteers were enrolled and screened using a Thai Stress Test (TST) to determine their stress status. Participants were divided into the stressed and the non-stressed groups. Synbiotics preparation comprised a mixture of probiotics strains in a total concentration of 1 × 10^10^ CFU/day (5.0 × 10^9^ CFU of *Lactobacillus paracasei* HII01 and 5.0 × 10^9^ CFU of *Bifidobacterium animalis* subsp. *lactis*) and 10 g prebiotics (5 g galacto-oligosaccharides (GOS), and 5 g oligofructose (FOS)). All parameters were measured at baseline and after the 12th week of the study. In the stressed group, the administration of synbiotics significantly (*p* < 0.05) reduced the negative scale scores of TST, and tryptophan. In the non-stressed group, the synbiotics administration decreased tryptophan significantly (*p* < 0.05), whereas dehydroepiandrosterone sulfate (DHEA-S), tumor necrosis factor-α (TNF-α), 5-hydroxyindoleacetic acid (5-HIAA), and short-chain fatty acids (SCFAs), acetate and propionate were increased significantly (*p* < 0.05). In both groups, cortisol, and lipopolysaccharide (LPS) were reduced, whereas anti-inflammatory mediator interleukin-10 (IL-10) and immunoglobulin A (IgA) levels were increased. In conclusion, synbiotics administration attenuated the negative feelings via the negative scale scores of TST in stressed participants by modulating the HPA-axis, IL-10, IgA, and LPS. In comparison, synbiotics administration for participants without stress did not benefit stress status but showed remodeling SCFAs components, HPA-axis, and tryptophan catabolism.

## 1. Introduction

Urbanization is a global phenomenon that transforms various aspects of everyday life. Living in a fast-paced environment drives post-traumatic stress disorder (PTSD) in parts of the vulnerable population, and its consequences can be psychotic related-events, stress-related illnesses, and/or depression [1]. The combination of the urban environment and stress exposure can alter the biochemical-related stress system. The stress response is governed by the hypothalamus-pituitary-adrenal axis (HPA-axis). Long-term stress exposure promotes HPA-axis hyperactivation, secreting cortisol that affects behavior, and promoting low-grade inflammatory cytokines secretion, which induces improper physical function systems to cause degenerative diseases of cardiovascular and neurodegeneration [2]. In addition, the dysregulation of the HPA-axis is associated with serious mental disorders such as major depressive disorder and schizophrenia [3]. The long-term perception of uncertainty stress correlates with the greater prevalence of mental disorders [4], and highly stressful work can also cause death without cardiometabolic conditions [5,6].

The HPA-axis activity has a connection to gut conditions. The collaboration between gut and brain, called the gut-brain axis, has been elucidated by four major pathways: neurologic, endocrine, humoral/metabolic, and immune [7]. The supplementation of probiotics or synbiotics could positively regulate the above-mentioned pathways and offer health benefits to the host [8].

According to World Health Organization/ Food and Agriculture Organization (WHO/FAO) 2002, probiotics have been defined as a living microorganism that benefits the host when administrated adequately [9]. Many clinical studies confirm the benefit of probiotics. According to the link between gut and brain on HPA-axis modulation, improvement in mental health is illustrated by a study on probiotics supplementation in preclinical and clinical studies. In the rat model study, the receiving of *Bifidobacterium infantis* could attenuate stress-like behavior and reduce corticosterone in mice [10]. In pregnant mice, the consumption of *Bifidobacterium animalis* subsp. *lactis* BB-12 and *Propionibacterium jensenii* 702 provoked stress process stimulation and enhanced stress tolerance in older adults [11]. Additionally, the consumption of *Lactobacillus farciminis* 10^11^ CFU/day for 12 weeks could attenuate low-grade inflammatory mediators IL-1β, IL-6, and LPS, finally restoring tight junction and reducing gut permeability [12].

Stress relief by probiotics intake has been the subject of academic study. The consumption of *L. casei* strain Shirota 1.0 × 10^11^ CFU per 100-mL bottle of fermented milk encouraged stress regulation in medical students before an examination. The test group showed lower expression of salivary cortisol than the placebo group, even though the salivary cortisol was not significantly changed throughout the intervention period [13].

Prebiotics are a non-digestible food ingredient that beneficially affects the host by selectively stimulating the growth and/or activity of one or a limited number of bacteria in the colon, thus improving host health [14]. Probiotics and prebiotics play a crucial role in physical and mental health manipulation [15,16]. The advantage of prebiotics administration on mental health has been proven in several studies [17]. Prebiotics oligosaccharides such as galacto-oligosaccharides (GOS) and fructo-oligosaccharide (FOS) have a positive effect on mental health, which has been proven in several studies [18]. The consumption of 5.5 g of GOS or FOS per day for 3 weeks reduced the waking salivary cortisol level in healthy subjects [19]. The studies suggested that the consumption of 7 g of trans-galacto-oligosaccharide improved mood and gut microbiota [20].

Furthermore, 5 g of FOS supplementation promoted a better stress index score, highlighting its role in improving anxiety [21]. Prebiotics oligosaccharides are a non-digestible carbohydrate and precursor for gut microbial metabolites short-chain fatty acids (SCFAs). The neuroprotective property is promoted by SCFAs upregulation activating G-protein coupled free fatty acid (FFAR) and/or by inhibiting histone deacetylase (HDAC) enzyme in the brain [22]. Oligosaccharides promote gut microbiota modification by enhancing commensal probiotics *Lactobacillus* and *Bifidobacterium* by suppressing infectious microbes like *Clostridium* [23].

Even though the probiotics and prebiotics administration are prone to deliver the benefits on stress regulation, the effects of both probiotics and prebiotics are still being debated [24]. Synbiotics is defined as a mixture comprising live microorganisms and substrate(s) selectively utilized by host microorganisms that confers a health benefit on the host [25].The subjects with moderate depression supplemented with a combination of *L. casei* (3 × 10^8^ CFU/g) *L. acidophilus* (2 × 10^8^ CFU/g), *L. bulgaricus* (2 × 10^9^ CFU/g), *L. rhamnosus* (3 × 10^8^ CFU/g), *B. breve* (2 × 10^8^ CFU/g), *B. longum* (1 × 10^9^ CFU/g), *S. thermophilus* (3 × 10^8^ CFU/g), and 100 mg FOS showed better Hamilton Depression Rating Scale (HAM-D) scores after 10 weeks of administration which referred to better mental health improvement [26].

The present study aimed to investigate the effects of the synbiotics supplement containing *L. paracasei* HII01, *B. animalis* subsp. *Lactis,* and GOS with FOS on the stress modulation in the stressed participants.

## 2. Materials and Methods

### 2.1. Study Design and Participants

The study consisted of two groups (the stressed and the non-stress group) compared at baseline and after synbiotics administration. The study design and protocol followed Good Clinical Practices and fully complied with the ethical guidelines of a clinical trial conducted under the Declaration of Helsinki. The Ethical Committee of the University of Phayao approved the study (Code: 1.3/005/64).

The previous study investigated the outcome of probiotics treatment on stress, mood, and anxiety in depression [27]. The stress score at the pre-treatment was 64.3 (standard deviation = 23.9). The outcome at post-treatment was 37.9 (standard deviation = 29.0). The stress score outcome with standard deviation, α-value = 0.05, correlation = 0.50, and power = 0.80 were calculated using the statistical software package STATA 15.1 (Statacorp, College Station, TX, USA). The estimated sample size with a 5% probable drop in the sample was 12 in each group. All participants of both groups were 18–65 years old males and females working and living in Chiang Mai province, who agreed with informed consent and whose stress status was diagnosed, following the Thai Stress Test (TST) guidelines [28]. The mild stress and stressful participants were defined as the stressed group, whereas the normal mental health and excellent mental health were defined as the non-stressed group identified by the Thai Stress Test (TST). The participants were non-malignant, free from vascular diseases, psychotic disorders, and drug and alcohol addiction. Moreover, all participants were asked to abstain from probiotics and prebiotics products during the study period.

**Figure 1:**
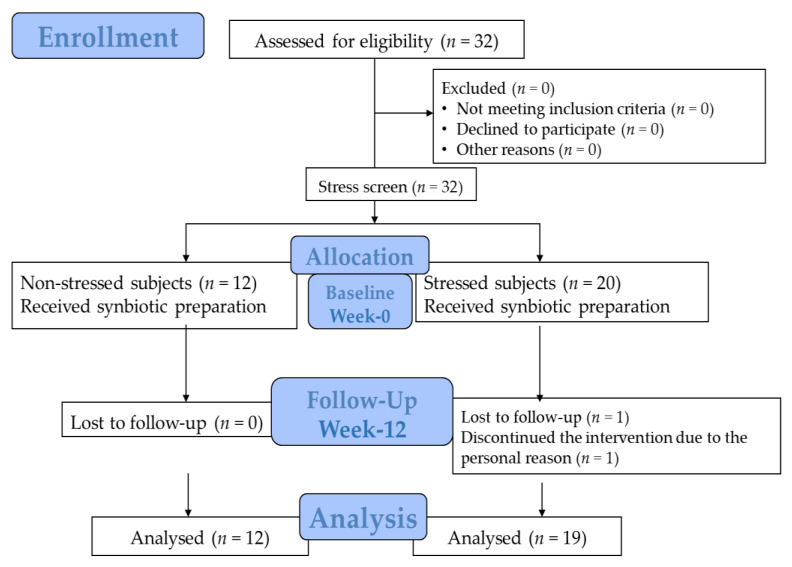
The allocation and follow-up chart.

The study was conducted for 12 weeks. All participants were asked to visit the research center twice at the beginning of the study and during its 12th week to collect the samples, monitor/follow up stress status, and make essential health assessments. Salivary, venous blood, first-morning urine, and stool samples were collected after 8 h of overnight fasting. The parameters were determined at the Associated Medical Science (AMS) Clinical Service Center, Faculty of Associated Medical Sciences, Chiang Mai University, Thailand. The study protocol is illustrated in Figure 1.

### 2.2. Synbiotics Administration

The synbiotics preparation used in this study comprised 2 probiotics strains and 2 prebiotics in an aluminum foil sachet. The synbiotics preparation is a mixture of probiotics strains in a total concentration of 10^10^ CFU (5.0 × 10^9^ CFU of *Lactobacillus paracasei* HII01 and 5.0 × 10^9^ CFU of *Bifidobacterium animalis* subsp. *lactis*) and 10 g prebiotics (5 g galactooligosaccharides (GOS), and 5 g oligofructose (FOS)). The probiotics were prepared and purchased from Lactomason Co., Ltd., Gyeongsangnam-do, South Korea. The prebiotics were purchased from New Francisco (Yunfu city) Biotechnology Cooperation Limited, Yunfu city, Guangdong, China (GOS; GOS-700-P), and Orafti^®^P95, (Beneo-Orafti S.A., Rue Louis Maréchal 1 4360 OREYE, Belgium) (FOS). The concentration of the probiotics strain was fixed according to the suggestions from the previous study [29].

Throughout the study, the participants were asked to consume the synbiotics preparation before breakfast every day by dissolving the content in water. The participants were on a dietary plan (as per the nutritionist’s suggestions, we provided 3 meals/day to the participants; 1200 calories per day; at the ratio of carbohydrate: protein: fat = 50:20:30) and sustained their routine medications and activities. Participants were asked to record their regular activities, and other additional medications and supplements during the study.

### 2.3. Outcome Measurements

The primary outcome measurements were stress status with negative and positive feelings manipulation after the 12th week of synbiotics administration. The secondary outcome measurements were the biomarkers of HPA-axis (salivary cortisol and dehydroepiandrosterone sulfate (DHEA-S)), neuro-inflammatory cytokines (tumor necrosis factor-α (TNF-α) and interleukin-10 (IL-10)), tryptophan metabolism (kynurenine pathway; tryptophan and kynurenine, serotonergic pathway; quinolinic acid (QA) and 5-hydroxyidoleacetic acid (5-HIAA)), gut microbial metabolites (short-chain fatty acids (SCFAs) and lipopolysaccharide (LPS)), and immunoglobulin A (IgA).

### 2.4. Participants’ Characteristic Data

Personal health data were collected, including eating habits, excretion, alcohol consumption, smoking, underlying diseases, medication, and regular supplementation. Demographic data, including sex, age, smoking habits, alcohol consumption, weight, and body mass index, were evaluated.

### 2.5. Stress Assessment

The study subjects were separated based on the results of the Thai Stress-test (TST). All the subjects tested for the Thai Stress Test were separated into two groups as detailed (Figure 1). The stress level was assessed via TST, developed by Phattharayuttawat et al. [28] for enrollment screening, measuring the stress status, scale scores at baseline, and the end of the study. TST has been assessed for construct validity and showed the Cronbach’s alpha coefficient of 0.84 and split-half coefficient of 0.88 [28]. The test comprised 24 questions, with 12 questions for negative feelings effects and 12 for positive feelings effects. Each question needs to be answered by participants based on their personal experience. The answers have a 3-rating scale: “0” means “never”, “1” means “sometimes”, and “3” means “often”.

Negative and positive question items were separately summarized. The matrix table has used both sides of the scale scores to identify the negative and positive scale score groups (Table 1). After grouping the negative and positive scale scores, the number in the matrix table showed the stress status. Therefore, the stress status was interpreted as follows: scoring group 1 was “Excellent mental health (if not faking)”, scoring groups 2, 3, and 4 were “Normal mental health,” scoring groups 5 and 6 were “Mild stress”, and scoring groups 7, 8, and 9 were “Stressful”.

### 2.6. HPA-Axis Assessment

Salivary samples were gathered at 7–9 a.m. for cortisol and DHEA-S measurement and stored at −20 °C before analysis. Both cortisol and DHEA-S were measured by enzyme-linked immunosorbent assay (ELISA) commercial kit (Eagle biosciences^®^, Amherst, NH, USA). Salivary cortisol ultrasensitive ELISA was for cortisol, and DHEA-S Saliva ELISA Assay Kit was for DHEA-S. The analysis method was followed according to the manufacturer’s instructions.

### 2.7. Neuroinflammatory Cytokines Assessment

Serum inflammatory cytokines TNF-α and IL-10 were determined using two commercial ELISA kits: The Human TNF-α ELISA kit and the IL-10 Human ELISA kit. The measurement protocol followed the instructions of the manufacturer Thermo fisher, Sydney, NSW, Australia.

### 2.8. Tryptophan Metabolism Assessment

Serum level tryptophan and kynurenine were analyzed via high-performance liquid chromatography (HPLC) following the modified method of Badawy and Morgan (2010). The sample was pushed into Synergi^TM^ 4 µL fusion-RP80 A column C18 (250 × 4.6 mm) at 37 °C with a 0.8 mL/min flow rate. Furthermore, 10 mM sodium dihydrogen phosphate (27:73 *v*/*v*) pH 2.8 was used as the mobile phase, and a UV detector at 220 nm was used [30].

The morning urine samples were collected for QA and 5-HIAA determination. The Human Quinolinic acid ELISA kit (Fivephoton Biochemicals^TM^, San Diego, CA, USA) was used for QA and 5-HIAA-ELISA-Kit-Urine-Fast-Track (Immusmol, Bordeaux, France) for 5-HIAA determination. The measurement protocols were followed according to manufacturer’s instructions.

### 2.9. Gut Microbial Metabolites and Immunoglobulin Assessment

The morning stool samples were used to determine SCFAs by HPLC, as detailed previously by Katoni et al. [31]. Shodex SH1011 column was used. A total of 5 mM sulfuric acid was used as the mobile phase with a flow rate of 0.6 mL/min [32].

Serum endotoxin LPS was analyzed by Human lipopolysaccharide (LPS) ELISA Kit (MyBioSource^®^, San Diego, CA, USA). Human IgA (Immunoglobulin A) ELISA kit (Elabscience^®^, Houston, TX, USA) was used for IgA determination.

### 2.10. Statistical Analyses

All statistical analyses were executed using STATA15.1 for Windows (Stata Corp, College Station, TX, USA). The license was for the Faculty of Pharmacy, Chiang Mai University, Chiang Mai, Thailand. All variable outcomes were displayed as mean ± standard of error (SE) and tested for normal distribution. Demographic data—age, weight, kidney function (Blood urine nitrogen (BUN) and creatinine clearance), liver function (Alanine aminotransferase (AST) and aspartate aminotransferase (ALT)), and serum cholesterol profile were assessed via *t*-test or Mann–Whitney U test, as appropriate. Sex, smoke habit, and alcohol consumption were evaluated via Fisher’s exact test. The different outcomes of each group at baseline and the end of the study were calculated using a paired *t*-test or Wilcoxon signed-rank test. The results between groups were compared using a *t*-test or Mann–Whitney U test as appropriate, and Gaussian regression was used to find the relation of both groups. All parameters were also evaluated by power analysis. Statistical significance was examined as a *p*-value less than 0.05.

## 3. Results

### 3.1. Characteristics of Participants

A total of 32 volunteers were enrolled. After the stress screening, 20 participants were classified as belonging to the stressed group, with 1 drop-out for personal reasons. The other volunteers, 12 participants, were identified as the non-stressed group. The demographic data of both groups were compared statistically and demonstrated no difference in all parameters (*p* < 0.05) (Table 2).

### 3.2. Effect of Synbiotics on Stress Status and Stress Score

**Figure 2:**
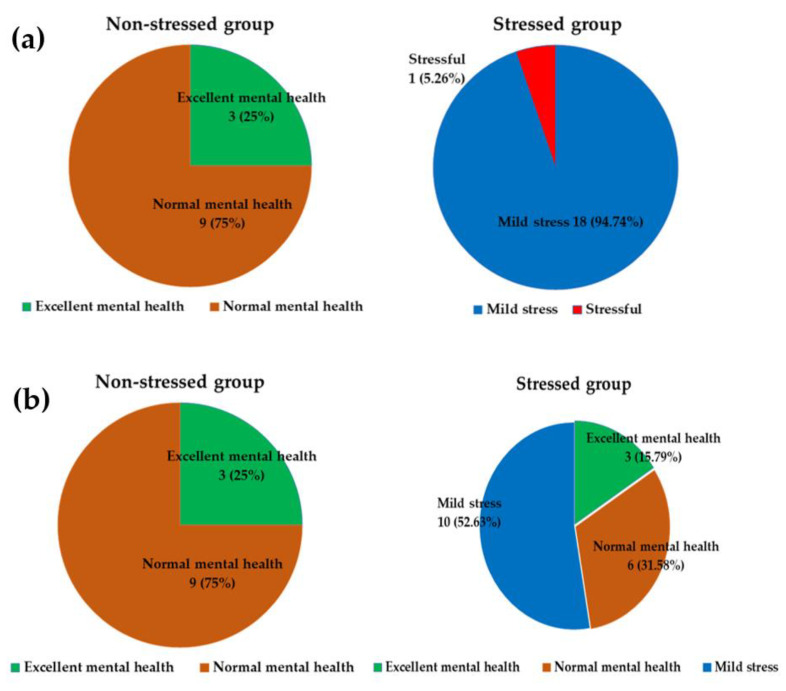
Stress status of the stressed and non-stressed groups (numbers of subjects (%)). The different color denotes each stress status. Green: Excellent mental health; Orange: Normal mental health; Blue: Mild stress; Red: Stressful. (**a**) Stress status at baseline, (**b**) Stress status after 12 weeks.

Stress status was identified as four statuses: excellent mental health (if not faking), normal mental health, mild stress, and stressful, according to Phattharayuttawat et al., 2000. At the baseline, the stress status of the stressed group was presented as 1 (5.26%) participant with stressful and 18 (94.74%) participants with mild stress. The non-stressed group comprised 9 (75%) participants with normal mental health and 3 (25%) participants with excellent mental health (if not faking), as shown in Figure 2a. The changes in stress status in stressed and non-stressed participants are displayed in Figure 2b. The synbiotics administration slightly improved stress status in the stressed group but not in the non-stressed group.

**Figure 3:**
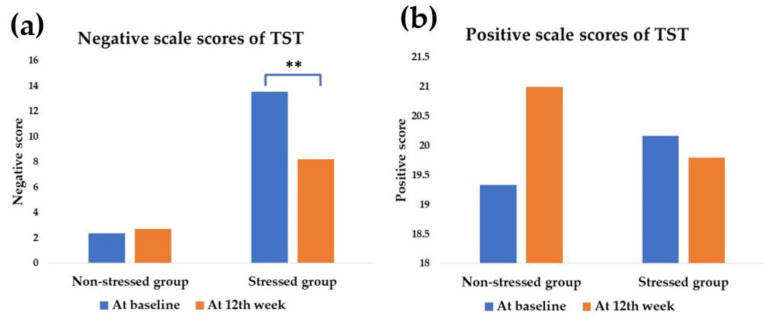
Modulation of mean TST scores according to either negative scale scores or positive scale scores after the synbiotics administration. The blue bar is the scores at the baseline, and the orange bar is the scores at the 12th week of synbiotics administration. ** significant difference (*p* less than 0.05). (**a**) The negative scale scores of TST in the stressed group were attenuated at the 12th week (*p* = 0.001, power = 0.98). (**b**) The positive scale scores of TST were not significantly changed in both groups (*p* < 0.05).

At baseline, the stressed group (13.53 ± 1.20) expressed a statistically significant high scale score on negative feelings compared with the non-stressed group (2.33 ± 0.54) (*p* < 0.001). In contrast, there was no statistical difference in the positive scale scores (Figure 3).

Although there was no significant change of stress status in the stressed group, the outcome was exhibited the decreasing negative scale scores of TST from 13.53 ± 1.20 at the baseline to 8.21 ± 1.33 at the 12th week; *p* = 0.001. In the meantime, there was no statistical difference in positive scale scores after the synbiotics administration in the stressed group. The positive scale scores of the stressed group were modified from 20.16 ± 1.59 to 19.79 ± 1.87, respectively (*p* = 0.668). In comparison, neither negative scale scores nor positive scale scores were modified from baseline in the non-stressed group after the 12th week of synbiotics administration. However, the negative scale scores in the non-stressed group were from 2.33 ± 0.54 to 2.67 ± 0.61, respectively (*p* = 0.418) and the positive scale scores were from 19.33 ± 2.62 to 21.00 ± 2.03, respectively (*p* = 0.668).

### 3.3. Effect of Synbiotics Administration on HPA-Axis and Inflammatory Cytokines

Before the synbiotics administration, the mean cortisol of the stressed participants and control exhibited no difference; however, a slightly higher level in the non-stressed group was observed (*p* < 0.05). At the same time, a high level of pro-inflammatory mediator TNF-α was found in the stressed group. It was modified from 89.75 ± 7.53 at baseline to 49.48 ± 5.07 ng/mL at the 12th week, respectively (*p* < 0.001).

**Figure 4:**
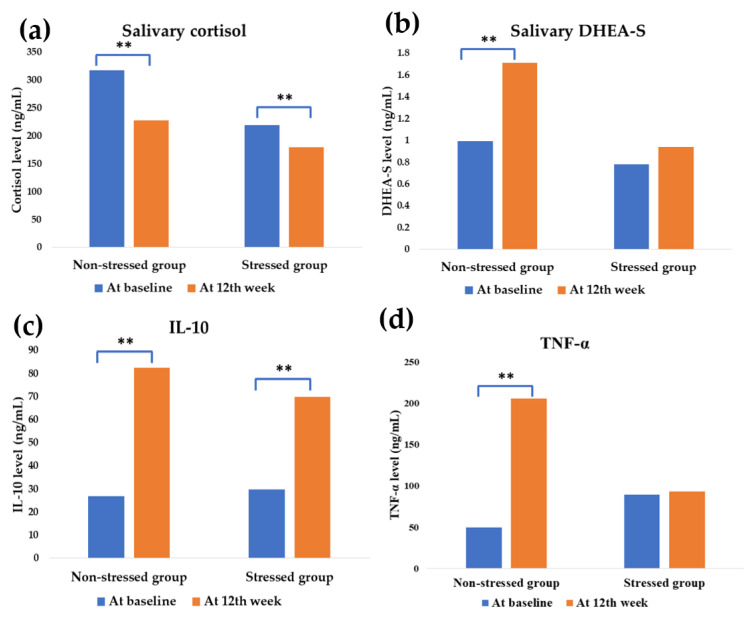
Effect of synbiotics administration on HPA-axis and neuro-inflammatory cytokines modulation. The blue and orange bar shows the values at baseline and the 12th week of synbiotics administration, respectively. ** significant difference (*p* less than 0.05). DHEA-S = Dehydroepiandrosterone sulfate; IL-10 = Interleukin-10; TNF-α = Tumor Necrosis Factor-alpha. The effect on HPA-axis: (**a**) Mean salivary cortisol value; stressed group: *p* = 0.033 (power = 0.65), non-stressed group: *p* = 0.015 (power = 0.96), (**b**) Mean DHEA-S value, non-stressed group: *p* = 0.012 (power = 0.99). The effect on inflammatory cytokines: (**c**) Mean IL-10 value; stressed group: *p* = 0.002 (power = 0.99), non-stressed group: *p* = 0.015 (power = 0.94), (**d**) Mean TNF-α value; non-stressed group: *p* = 0.002 (power = 0.99).

After the administration, HPA-axis was downregulated in both groups by attenuating cortisol levels (*p* < 0.05). The synbiotics also induced DHEA-S upregulation in the non-stressed group (*p* < 0.05), as shown in Figure 4.

Salivary cortisol was improved from 219.37 ± 29.13 ng/mL at baseline to 154.77 ± 7.74 ng/mL (*p* = 0.033) in the stressed group and 275.24 ± 17.80 ng/mL at baseline to 204.16 ± 17.18 ng/mL (*p* = 0.015) in the non-stressed group. DHEA-S was also modulated from 0.99 ± 0.14 at baseline to 1.71 ± 0.23 ng/mL at the 12th week (*p* = 0.012) in the non-stressed group.

Synbiotics promoted inflammatory mediation at the end of the study. The anti-inflammatory IL-10 was significantly upregulated in both stressed and non-stressed participants. In the stressed group, IL-10 was modified from 29.82 ± 0.70 to 69.84 ± 9.44 ng/mL at the 12th week (*p* = 0.002) and the modification was found from 26.94 ± 1.85 to 82.32 ± 15.07 ng/mL, (*p* = 0.015) for the non-stressed group. Interestingly, the proinflammatory cytokine TNF-α was significantly increased in the non-stressed group from 49.48 ± 5.07 at the baseline to 205.74 ± 33.86 ng/mL at the 12th week (*p* = 0.002). In the meantime, there was no modification found in the stressed group.

### 3.4. Effect of Synbiotics Administration on Tryptophan Metabolism

At baseline, the precursor tryptophan of the stressed group displayed lower than the non-stressed group (from 26.86 ± 1.62 to 33.64 ± 1.54 µmol/L; *p* = 0.008, respectively) and also with the catabolite kynurenine (from 1.11 ± 0.22 to 1.54 ± 0.13 µmol/L; *p* = 0.033, respectively) whereas QA (6.03 ± 0.42 and 5.83 ± 0.23 ng/mL; *p* = 0.712, respectively) and 5-HIAA (3.25 ± 0.52 and 2.34 ± 0.18 mg/L; *p* = 351, respectively) displayed a higher tendency.

The tryptophan metabolism was expressed in the difference after the synbiotics administration between groups. The precursor tryptophan was significantly downregulated in both stressed and non-stressed group (from 26.86 ± 1.62 to 21.67 ± 3.57 µmol/L, *p* = 0.003 and from 33.64 ± 1.54 to 22.94 ± 1.08 µmol/L, *p* < 0.001, respectively).

**Figure 5:**
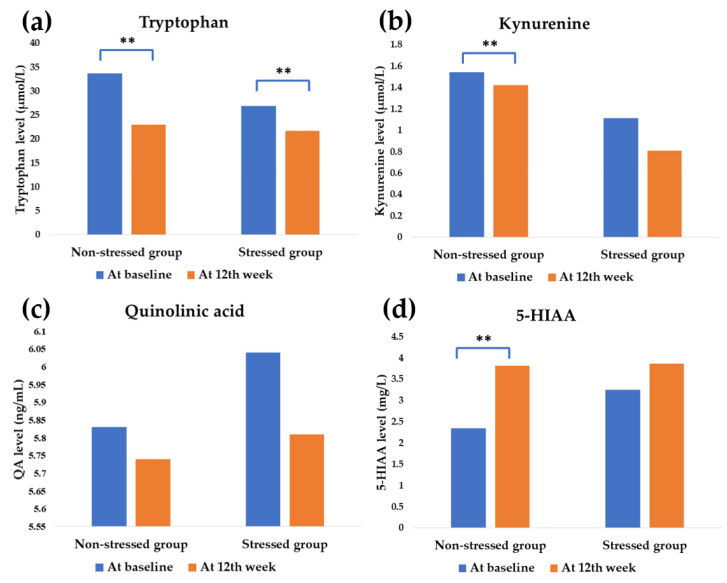
Effect on tryptophan metabolites. The blue and orange bar shows the values at baseline and the 12th week of synbiotics administration, respectively. ** significant difference (*p* less than 0.05). 5-HIAA = 5-Hydroxyindoleacetic acid. The tryptophan modulation on kynurenine pathway: (**a**) Mean tryptophan value: stressed group, *p* = 0.003 (power = 0.94), non-stressed group, *p* < 0.001 (power = 1.00), (**b**) Mean kynurenine value. The modulation of tryptophan metabolism to the serotonergic pathway: (**c**) Mean quinolinic acid value, (**d**) Mean 5-HIAA value: non-stressed group, *p* < 0.001 (power = 1.00).

In the non-stressed group, the tryptophan was metabolized to convert all parameters at the end of the study, especially 5-HIAA. It was modulated from 2.34 ± 0.18 to 3.81 ± 0.26 mg/L, (*p* < 0.001), respectively. In comparison, there was no statistical modification found for kynurenine and QA (*p* < 0.05) (Figure 5)

### 3.5. Effect of Synbiotics Administration on Gut Microbial Metabolites and Immunoglobulin

Before synbiotics administration, SCFAs propionate and butyrate expressed a statistical difference. Propionate in the stressed group displayed a higher level than the non-stressed (19.99 ± 2.04 and 11.40 ± 1.57 mmol/g; *p* = 0.006, respectively) while butyrate was lower (6.38 ± 0.40 and 7.56 ± 0.24 mmol/g; *p* = 0.036, respectively). A significant difference was not observed in either LPS or IgA.

**Figure 6:**
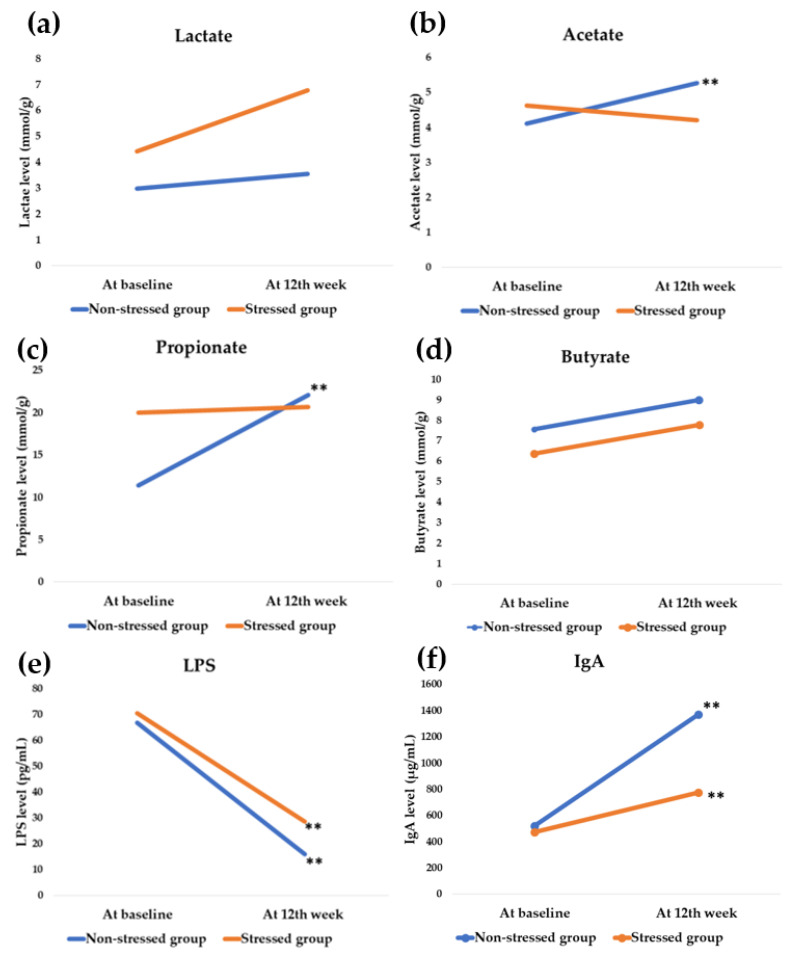
Effect on gut microbial metabolites. The blue and orange bar shows the values at baseline and the 12th week of synbiotics administration, respectively. ** significant difference (*p* less than 0.05). LPS = Lipopolysaccharide; IgA = Immunoglobulin A. Modulation of SCFAs. (**a**) Mean change of lactate, (**b**) Mean change of acetate: non-stressed group: *p* < 0.001 (power = 1.00), (**c**) Mean propionate value: non-stressed group: *p* < 0.001 (power = 1.00), (**d**) Mean change of butyrate value. The modulation of endotoxin: (**e**) Mean change of LPS value: stressed group: *p* < 0.001 (power = 0.97), non-stressed group: *p* = 0.002 (power = 0.92). Modulation of immunoglobulin: (**f**) Mean IgA value: stressed group; *p* = 0.004 (power = 0.81), non-stressed group: *p* = 0.003 (power = 1.00).

Unexpectedly, there was no statistical modification of SCFAs in the stressed group at the end of the study. In contrast, a significant modulation of acetate (4.11 ± 0.10 to 5.26 ± 0.24 mmol/g; *p* < 0.001) and propionate (11.40 ± 1.57 to 22.06 ± 0.52 mmol/g; *p* < 0.001) was found in the non-stressed group as shown in Figure 6.

Synbiotics attenuated LPS level in both stressed participants (70.35 ± 11.43 to 28.57 ± 8.84 pg/mL, *p* < 0.001) and non-stressed participants (66.78 ± 13.73 to 16.01 ± 3.74 pg/mL, *p* = 0.002), whereas it promoted IgA elevation. In the stressed group, IgA was elevated from 473.79 ± 56.80 to 773.17 ± 114.76 µg/mL (*p* = 0.004) and from 521.14 ± 65.29 to 1367.71 ± 107.15 µg/mL (*p* = 0.003) in the non-stressed group.

### 3.6. Differences in Pre- and Post-Administration Status among the Stressed and the Non-Stressed Groups

The pre-and post-administration differences showed a significant decrease in the tryptophan in the stressed and the non-stressed groups (*p* < 0.05; power = 0.77). The non-stressed group displayed higher DHEA-S levels but slightly lower than the stressed group (*p* < 0.05, power = 0.62).

The IgA, acetate, and propionate levels showed a statistical difference in the stressed and non-stressed groups. The IgA level increased significantly in both groups (*p* < 0.05; power = 0.98). Nonetheless, a higher propionate increase was observed in the non-stressed group (*p* < 0.05; power = 0.84). Unexpectedly, the negative (power = 0.94) and positive (power = 0.94) scale scores of the TST slightly increased in the non-stressed groups and decreased in the stressed group (*p* < 0.05) (Table 3).

### 3.7. Gaussian Regression Analysis of the Outcomes at the End of the Study

The Gaussian regression analysis of the outcomes after the 12th week of the synbiotics administration indicated that the synbiotics administration significantly modified cortisol level (power = 0.92) but downregulated IL-10 (power = 0.82). The other parameters were not altered significantly (Table 4).

## 4. Discussion

Stress exposure affects the gut–brain axis [33]. Stress manipulation by probiotics and prebiotics, particularly GOS and FOS, has been proven in numerous studies [34,35,36]. In clinical studies, the probiotics strain *L. paracasei* HII01 positively affects cholesterol, obesity, and diabetes indices modulation [37,38]. However, this study investigated the greater benefit of stress manipulation of *L. paracasei* HII01 using a synbiotics formula. The study population was small in the present study. The power analysis of each parameter was calculated, and the values are higher than 0.80 (Figure 3, Figure 4, Figure 5 and Figure 6), except salivary cortisol level in the stressed group.

Our mental health status depends on our capacity for handling our feelings. Positive feelings include happiness and life satisfaction; on the other hand, negative feelings include sadness and life dissatisfaction. If someone has more negative feelings than positive thoughts, we consider them in disturbed mental states or stressed mental states [39].

Regarding stress evaluation tools, negative feelings are the most abundant. Accordingly, the appropriate tools for stress assessment should evaluate both sides of feelings. One of the common tools, such as the Perceived stress scale-10, assessed perceived distress and the ability to cope with stress [40]. In comparison, the General Health Questionnaire (GHQ) Thai version inspects the perceived stress on mental and physical symptoms [41]. Conversely, the TST is the tool to evaluate both feelings of the mind (the negative and positive feelings) corresponding with psychological well-being [28].

In this study, the stress status of the participants was classified based on TST scores. The stressed subjects had more negative scale scores and non-significant positive scale scores at baseline. We may conclude that the stressed subjects might have poor stress management ability. After the synbiotics administration, the negative scale scores were significantly reduced, as mentioned in Figure 3. The results suggest that synbiotics administration could attenuate negative feelings in stressed participants.

Stress response corresponds to the HPA-axis, which is particularly associated with cortisol level [42]. During a stressed condition, the cortisol level increases and tries to regulate stress. However, hypercortisolemia does not mean that the subjects have excellent stress regulation. It is a sign of chronic fatigue syndrome when cortisol levels drop after acute elevation [43]. At the baseline, the cortisol level was lower in stressed subjects. No matter the cortisol level in both groups, the synbiotics administration promoted cortisol lowering effects significantly.

Synbiotics administration was found to stimulate the secretion of the anti-inflammatory cytokine IL-10 in stressed and non-stressed groups. Elevated IL-10 promotes HPA-axis modulation via adrenocorticotrophic hormone (ACTH) inhibition [44]. The low level of IL-10 is associated with prolonged stress exposure and depressive-like behavior [45]. Despite IL-10 providing an advantage for mental health modulation, the modification was found only in stressed participants. Conversely, elevated IL-10 exhibited no effect on mental modulation in the normal mental health in the non-stressed group.

Synbiotics administration promoted the TNF-α level, which mediates the indoleamine-2,3-oxygenase enzyme (IDO) [46] in tryptophan catabolism. TNF-α elevation was observed in the non-stressed but not the stressed participant group. Tryptophan is an essential amino acid correlated with mental health in animals and humans. The metabolites of tryptophan play a pivotal role in neurodegenerative diseases and mental health [47].

Surprisingly, synbiotics administration does not affect tryptophan metabolism in stressed subjects. Usually, the neurotoxic pathway originates from the catabolism of tryptophan by the IDO enzyme. Kynurenine, the metabolite of this reaction, is associated with neuropsychiatric symptoms [48]. Therefore, the several reactions after the kynurenine conversion induce QA production which is a known neurotoxin; moreover, it also induces oxidative stress, glutamine excitotoxicity, and mitochondria dysfunction [49].

Conversely, the neuroprotective route of tryptophan catabolism is via several enzymes such as monoamine oxidase (MAO) and aldehyde dehydrogenase to produce 5-HIAA, which is indicated to be a biomarker of neuropsychiatric disorders [50]. As per the results, it was found that the negative feelings attenuation of synbiotics administration might not occur via tryptophan metabolism modulation. In comparison, the low level of tryptophan and the increase of 5-HIAA level in the non-stressed group demonstrated no effect on stress status and scale scores (Figure 5).

A connection between stress regulation and gut commensal microbial metabolites has been suggested [51,52]. SCFAs such as propionate and butyrate play a role in neuroprotective and psychiatric disorder treatment [53,54]. According to the preclinical study of Maltz et al. (2019), low levels of acetate and butyrate were observed in stressed mice [55]. We observed that stressed subjects showed higher propionate levels than the non-stressed group. Unexpectedly, the effect of synbiotics on the SCFAs in the stressed participants showed no statistical difference (Figure 6). According to this result, the negative feelings modulation may not occur via SCFAs.

The gut microbial endotoxin LPS correlates with neuropsychiatric disorders [56]. LPS, by itself, can cross the blood-brain barrier and affect neurophysiology, which in turn affects emotion and behavior [57]. The rising LPS level is accompanied by negative emotion, anxiety, social disconnection, anhedonia, and fatigue [58]. LPS can influence glucocorticoid receptor expression in the hypothalamus that involves HPA-axis regulation [59,60,61]. The downregulation of LPS according to the synbiotics administration in the current study corresponded to the negative scale scores of TST.

The IgA is an antibody found abundantly in the intestinal epithelial and plays a role in antimicrobial activity, regulating the healthy composition and metabolic function of gut microbiota [62,63]. In addition, the alteration of IgA is also associated with stressful life events [64]. Psychological and physical stressors have a negative effect on IgA generation [65,66]. The results of this study indicate that the negative scale scores of TST were influenced by IgA elevation.

In summary, consumption of synbiotics improved negative feelings in stressed participants. According to our findings, the possible action is governed by modulating the HPA-axis, IL-10, LPS, and IgA. It is proven via the modulation of the negative scale scores at baseline after the synbiotics administration (Figure 3).

## 5. Conclusions

The current study was conducted with a small number of experimental subjects, and the results have to be confirmed again with extended studies. Nonetheless, the results showed that synbiotics administration reduced negative feelings in stressed participants. Furthermore, the synbiotics administration altered the HPA-axis, IL-10, LPS, and IgA levels.

Further studies are required on how the provided synbiotics administration alters the microbiota to discover the mechanism behind the positive effect of synbiotics on stress status.

## Figures and Tables

**Table 1:** Matrix table for the index of TST [28].

Negative Scale Score (Sum of Item 1–12)	Positive Scale Score				
	(Sum of Item 13–24)				
	12–36	9–11	6–8	3–5	0–2
0–1	1	2	3	4	5
2–3	2	3	4	5	6
4–5	3	4	5	6	7
6–7	4	5	6	7	8
8–36	5	6	7	8	9

**Table 2:** Characteristics of participants at the baseline.

Parameters	Non-Stressed Group (*n* = 12)	Stressed Group (*n* = 19)	*p*-Value *
Male, *n* (%)	3 (25.00)	8 (42.11)	0.282 ^c^
Female, *n* (%)	9 (75.00)	11 (57.89)	
Age (years)	43.17 ± 3.01	45.79 ± 3.10	0.572 ^a^
Weight, kg	75.20 ± 4.28	76.75 ± 3.64	0.788 ^b^
Body mass index (BMI), kg/m^2^	29.70 ± 1.59	29.36 ± 1.28	0.872 ^b^
Blood urea nitrogen (BUN), mg/dL	12.33 ± 1.19	13.32 ± 0.80	0.481 ^a^
Creatinine clearance, mg/dL	0.90 ± 0.11	0.87 ± 0.05	0.810 ^a^
Aspartate aminotransferase (AST), IU/L	25.17 ± 6.01	24.37 ± 5.62	0.776 ^a^
Alanine aminotransferase (ALT), IU/L	29.17 ± 9.04	27.63 ± 6.96	0.855 ^b^
Smoking, *n* (%)			
No	12 (100.00)	19 (100.00)	-
Yes	0(0.00)	0(0.00)	
Alcohol drinking, *n* (%)			
No	11 (91.67)	13 (68.42)	0.143 ^c^
Yes	1 (8.33)	6 (31.58)	

* *p*-value at 95% confidence interval. ^a^ *p*-value was calculated from the *t*-test. ^b^ *p*-value was calculated from Mann-Whitney U test. ^c^ *p*-value was calculated from Fisher’s exact test.

**Table 3:** Comparison of the differences between pre- and post-administration.

Parameters	Differences between Pre- and Post-Administration		
	Non-Stressed Group	Stressed Group	*p*-Value *
TST scores			
Negative scale scores ^#^	0.33	−5.32	0.009 ^b^**
Positive scale scores ^#^	0.33	−5.32	0.009 ^b^**
HPA-axis			
Cortisol (ng/mL)	−89.48	−40.34	0.491 ^b^
DHEA-S (ng/mL)	0.73	−0.01	0.004 ^b^**
Inflammatory cytokines			
IL-10 (ng/mL)	55.38	40.02	0.256 ^b^
TNF-α (ng/mL)	156.27	3.28	<0.001 ^b^**
Tryptophan metabolism			
Tryptophan (µmol/L)	−10.7	−5.20	0.029 ^b^**
Kynurenine (µmol/L)	−0.12	−0.30	0.919 ^b^
QA (ng/mL)	−0.09	−0.23	0.824 ^b^
5-HIAA (mg/L)	1.47	0.61	0.626 ^b^
Gut microbial metabolites & SCFAs			
Lactate (mmol/g)	0.55	2.34	0.351 ^b^
Acetate (mmol/g)	1.14	1.31	0.016 ^b^**
Propionate (mmol/g)	10.66	0.64	0.007 ^b^**
Butyrate (mmol/g)	1.42	1.39	0.208 ^b^
Endotoxin			
LPS (pg/mL)	−50.77	−41.78	0.394 ^b^
Immunoglobulin			
IgA (µg/mL)	846.57	299.37	<0.001 ^b^**

* *p*-value at 95% confidence interval, ** significant difference (*p* less than 0.05). ^#^ Mean of negative and positives scale scores of Thai Stress Test (TST). ^b^ *p*-value was calculated from Mann-Whitney U test; DHEA-S = Dehydroepiandrosterone sulfate; IL-10 = Interleukin-10; TNF-α = Tumor Necrosis Factor-alpha; QA = Quinolinic acid; 5-HIAA = 5-Hydroxyindoleacetic acid; LPS = Lipopolysaccharide; IgA = Immunoglobulin A.

**Table 4:** Gaussian regression analysis of the outcomes after the 12th week.

Parameters	Coefficient	95% CI	*p*-Value *
TST score			
Negative scale scores ^#^	2.39	−3.06 to 7.84	0.372
Positive scale scores ^#^	0.77	−8.04 to 7.09	0.897
HPA-axis			
Cortisol (ng/mL)	−72.63	−120.31 to −24.96	0.005 **
DHEA-S (ng/mL)	−0.85	−1.73 to 0.03	0.057
Inflammatory cytokines			
IL-10 (ng/mL)	−33.27	−84.60 to 18.08	0.192
TNF-α (ng/mL)	−79.85	−176.28 to 16.58	0.100
Tryptophan metabolism			
Tryptophan (µmol/L)	0.67	−4.63 to 4.96	0.942
Kynurenine (µmol/L)	−0.26	−0.77 to 0.24	0.289
QA (ng/mL)	−0.18	−1.06 to 0.70	0.670
5-HIAA (mg/L)	0.65	−1.17 to 2.47	0.466
Gut microbial metabolites & SCFAs			
Lactate (mmol/g)	3.71	−1.32 to 8.75	0.139
Acetate (mmol/g)	−1.19	−2.18 to 0.16	0.082
Propionate (mmol/g)	−1.07	−9.69 to 7.55	0.798
Butyrate (mmol/g)	−1.17	−9.87 to 7.53	0.782
Endotoxin			
LPS (pg/mL)	4.25	−26.21 to 34.72	0.774
Immunoglobulin			
IgA (µg/mL)	−554.95	−1037.47 to −72.43	0.026 **

* *p*-value at 95% confidence interval, ** significant difference (*p* less than 0.05). ^#^ Mean of negative and positive scale scores of Thai Stress Test (TST). DHEA-S = Dehydroepiandrosterone sulfate; IL-10 = Interleukin-10; TNF-α = Tumor Necrosis Factor alpha; QA = Quinolinic acid; 5-HIAA = 5-Hydroxyindoleacetic acid; LPS = Lipopolysaccharide; IgA = Immunoglobulin A.

## Data Availability

Data is contained within the article.
